# Emerging Biosensors to Detect Severe Acute Respiratory Syndrome Coronavirus 2 (SARS-CoV-2): A Review

**DOI:** 10.3390/bios11110434

**Published:** 2021-11-02

**Authors:** Wei Yin Lim, Boon Leong Lan, Narayanan Ramakrishnan

**Affiliations:** Electrical and Computer Systems Engineering, School of Engineering and Advanced Engineering Platform, Monash University Malaysia, Bandar Sunway 47500, Selangor, Malaysia; WeiYin.Lim@monash.edu (W.Y.L.); lan.boon.leong@monash.edu (B.L.L.)

**Keywords:** biosensor, COVID-19 diagnosis, SARS-CoV-2, surface plasmon resonance, field-effect transistor, electrochemical, a point-of-care device

## Abstract

Coronavirus disease (COVID-19) is a global health crisis caused by the severe acute respiratory syndrome coronavirus 2 (SARS-CoV-2). Real-time reverse transcriptase-polymerase chain reaction (RT-PCR) is the gold standard test for diagnosing COVID-19. Although it is highly accurate, this lab test requires highly-trained personnel and the turn-around time is long. Rapid and inexpensive immuno-diagnostic tests (antigen or antibody test) are available, but these point of care (POC) tests are not as accurate as the RT-PCR test. Biosensors are promising alternatives to these rapid POC tests. Here we review three types of recently developed biosensors for SARS-CoV-2 detection: surface plasmon resonance (SPR)-based, electrochemical and field-effect transistor (FET)-based biosensors. We explain the sensing principles and discuss the advantages and limitations of these sensors. The accuracies of these sensors need to be improved before they could be translated into POC devices for commercial use. We suggest potential biorecognition elements with highly selective target-analyte binding that could be explored to increase the true negative detection rate. To increase the true positive detection rate, we suggest two-dimensional materials and nanomaterials that could be used to modify the sensor surface to increase the sensitivity of the sensor.

## 1. Introduction

COVID-19 is an infectious disease caused by the severe acute respiratory syndrome coronavirus 2 (SARS-CoV-2). COVID-19 was first reported in Wuhan, China in December 2019 and spread rapidly across the world [1,2]. On 11 March 2020, the World Health Organization (WHO) declared the global spread of the novel coronavirus a pandemic and alerted the world to prepare for widespread community transmission [3]. Unfortunately, the transmission rate of SARS-CoV-2 is greater than the coronavirus for the severe acute respiratory syndrome (SARS) and the Middle East respiratory syndrome (MERS) in 2002 and 2012, respectively. COVID-19 was the leading cause of death in 2020 owing to the highly contagious and pathogenic coronavirus SARS-CoV-2. Although various vaccines have been developed and administered in various countries, the pandemic is still ongoing due to the emergence of multiple SARS-CoV-2 variants, particularly the Delta variant, which is much more transmissible. Up to 31 July 2021, there have been more than 197,252,280 confirmed COVID-19 cases globally, with 4,210,452 associated deaths [4].

The SARS-CoV-2 virus rapidly multiplies in the body tissues and triggers the immune system. The symptoms of COVID-19 may appear 2–14 days after exposure to the virus, ranging from mild fever to severe symptoms requiring hospitalization, such as difficulty breathing or shortness of breath [5]. To date, several biomarkers can be used for the detection of SARS-CoV-2: viral nucleic acid (single-stranded ribonucleic acid, RNA), viral protein antigen (spike (S) or nucleocapsid (N)) and antibodies (IgM, IgG or IgA) [6,7]. In general, diagnostic tests for COVID-19 fall into two main categories: viral tests that detect viral nucleic acid and protein antigens, and serological tests that detect anti-SARS-CoV-2 immunoglobulins [8]. The viral nucleic acid and antigen detection tests are used to assess the early stages of active infection, while the antibody tests provide evidence of the previous infection (recovery phase) [9].

Real-time reverse transcription-polymerase chain reaction (RT-PCR) targeting the SARS-CoV-2 RNA is the reference standard diagnostic test for COVID-19. The method is based on the reverse transcription of the viral RNA into complementary DNA (cDNA), followed by isothermally amplifying the specific regions of cDNA and detection by quantitative RT-PCR [10,11]. Although the RT-PCR test has high true positive and true negative detection rates, it is laborious, which leads to a long turn-around time from sample collection to test results. The lengthy RNA isolation steps (which takes approximately 2–4 h) requires highly trained manpower. RT-PCR testing becomes challenging if there are reagent shortages and a large number of samples. PCR tests are therefore not suitable for remote or resource-limited settings. Moreover, false negatives may arise if the sample is collected before the onset of symptoms or due to inadvertent contamination of reagent or specimen [12,13,14].

To address these limitations, rapid diagnostic tests based on antigen or antibody detection in respiratory samples (e.g., sputum, saliva, throat swab) or blood can provide timely detection at or near the point of care (POC) without the need for sophisticated laboratory facilities. These rapid tests have numerous benefits over the laboratory test, including rapidity of results (within 30 min), lower cost, easy to operate and suitable for large-scale screening outside the laboratory without the need for specialist operators [15,16,17]. Rapid antigen test is useful for early detection of active infection with a high viral load of SARS-CoV-2. The viral antigen usually appears within a few days after the onset of symptoms before the production of antibodies. A rapid antibody test is more suitable during the convalescent phase of the disease. The prevalence of antibodies in the human blood serum of individuals suspected of being infected with SARS-CoV-2 may take several days or weeks to develop after exposure to the virus. Immunoglobulin M (IgM) will be produced after three to six days of infection and immunoglobulin G (IgG) will be detectable after eight days of infection [18,19]. The Board Decision on Additional Support for Country Responses to COVID-19 has approved 47 and 48 types of rapid SARS-CoV-2 antibody and antigen diagnostic tests, respectively, for home use [20]. However, although these rapid POC tests have high true negative rates, the true positive rates are lower than the RT-PCR test [15,21,22]. The WHO acceptable minimum true positive and true negative rates are 80% and 97%, respectively [23].

Biosensors, which satisfy the WHO’s ASSURED criteria: Affordable, Sensitive, Specific, User-friendly, Rapid and Robust, Equipment-free, and Deliverable to end-users [24,25,26], are potential alternatives to the rapid POC antigen and antibody tests. A biosensor is an analytical device consisting of a biological element (e.g., nucleic acids, enzymes, antibodies, whole cells, or receptors) combined with a transducer for the detection of an analyte. The biological element binds the analyte of interest to the biosensor, and the transducer measures the interaction between the analyte and recognition element and converts the output to a measurable signal proportional to the analyte concentration [27,28,29,30]. With the integration of a suitable microfluidic platform and necessary mechanical enclosure, a biosensor can be translated into a POC system, where the sample collection and testing can be performed in the same device [31].

A variety of biosensors have been developed for real-time diagnostic of COVID-19, including surface plasmon resonance (SPR)-based biosensors, electrochemical biosensors and field-effect transistor (FET)-based biosensors. These biosensors have been successfully tested in proof-of-concept studies. However, none of the sensors have been translated into a POC device for commercial use yet. In this paper, we review the most recently developed SPR-based, electrochemical, and FET-based biosensors for SARS-CoV-2 detection. We explain how these sensors work, and discuss their advantages and limitations. Finally, we propose strategies to enhance the sensing performance of these biosensors to meet the WHO minimum requirement for true positive and negative detection rates.

## 2. Latest Developed Biosensors for COVID-19

**Figure 1:**
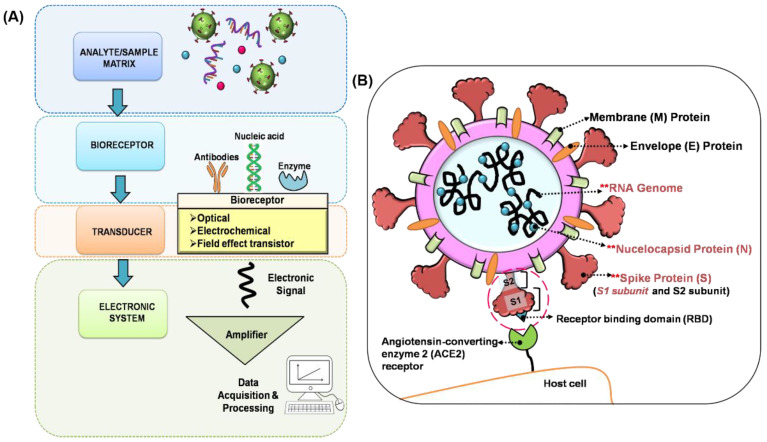
(**A**) Schematic diagram of a biosensor. (**B**) Structure and function of SARS-CoV-2 virus. The main four viral surface proteins are Spike (S), Envelope (E), Membrane (M) and nucleocapsid (N). S protein contains the receptor-binding domain (RBD) that recognises the host cell receptor, i.e., the human angiotensin-converting enzyme 2 (ACE2). E protein contributes to the assembly and morphogenesis of virions. M protein, which is embedded in a lipid bilayer, is responsible for the release of nutrients at the transmembrane and form the viral envelope. N protein binds to the RNA genome to form the nucleocapsid. **Preferred target analyte used for current COVID-19 biosensor development.

Compared to time staking laboratory tests, a biosensor with fast response and high accuracy in the form of a point-of-care device would be an excellent aid for the early diagnosis of SARS-CoV-2 infection. As shown in Figure 1A, generally a biosensor comprises of biosensing elements such as nucleic acids, enzymes, or antibodies that can interact with the target analyte in the form of biological or chemical sample, a transducer that can convert the changes in the biosensing element to an electrical signal, and an amplifier and necessary electronic system to process the electrical signal to digitise the output [27,32]. The COVID-19 outbreak has spurred the development of various types of biosensors with different biosensing platforms that can be used for the diagnosis of COVID-19.

SARS-CoV-2 contains four structural proteins, spike (S), membrane (M), envelope (E) and nucleocapsid (N) proteins, as shown in Figure 1B. The S protein is composed of two subunits S1 and S2, which are responsible for the attachment, fusion and entry of the virus. The S1 subunit contains a receptor-binding domain (RBD) that recognises host cell receptors and human angiotensin-converting enzyme 2 (ACE2). The S2 subunit facilitates membrane fusion for virus entry. N proteins bind to the viral RNA genome to form the nucleocapsid. M proteins release nutrients at the transmembrane and form the viral envelope. E proteins are small polypeptides that are responsible for the assembly and release of the virus. The SARS-CoV-2 viral RNA genome consists of open reading frames (ORFs) genes, which encode a total of 16 non–structural proteins (e.g., ORF1ab gene), and at least four structural proteins (e.g., S gene, E gene, M gene, N gene) [33,34]. For SARS-CoV-2 detection, three types of diagnostic tests—on nasopharyngeal swab, throat swab, blood and saliva samples—have been widely used based on (i) virus detection—hybridisation between sequence complementary (capture probe) to the target RNA genome, (ii) antigen protein detection—interaction of monoclonal antigen-specific antibody and virus antigen protein and (iii) antibody detection—interaction of recombinant antigen and target neutralising antibody. As described in the following sections, the current development of biosensors employs these approaches to target SARS-CoV-2 RNA genome (ORF1ab gene, RNA-dependent RNA polymerase (RdRP) gene, S gene, N gene) [35,36,37,38], specific antigen protein (S protein (S1 subunit/RBD), N protein) [39,40,41,42] and neutralising antibody (IgM, IgG) [35,43].

### 2.1. Methodology Used in Review Process

We followed the Preferred Reporting Items for Systematic Review and Meta-Analysis (PRISMA) guidelines for the review process. We searched the Web of Science and LENS. ORG databases using these keywords: COVID-19, SARS-CoV-2 and Biosensor, for articles dated from 1 January 2020 to 17 May 2021. A total of 439 articles were retrieved from this comprehensive search. Removal of duplicate and non-journal articles reduced the total to 297 articles. Subsequent screening excluded 143 review articles. Further exclusion of articles not related to three well-established biosensors: surface plasmon resonance, electrochemical and field-effect transistor-based sensors, reduced the total to 15 articles. We require at least experimental proof of concept of SARS-CoV-2 detection for the SPR-based, electrochemical and FET-based biosensors; therefore, we did not further exclude articles that did not report the validation of the sensors in the lab, i.e., report true positive and negative detection rates.

In the following sections, we explain how the three biosensors work and discuss their advantages and limitations. Table 1 presents the summary of these latest biosensors.

### 2.2. Surface Plasmon Resonance (SPR)/Localised Surface Plasmon Resonance (LSPR) Biosensor

**Figure 2:**
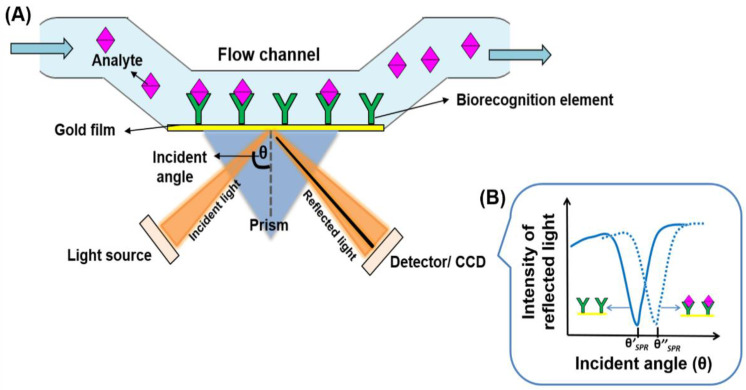
(**A**) Typical Surface Plasmon Resonance (SPR) configuration, (**B**) Resonance angle after antibody immobilisation on sensor surface (θ′_SPR_) and after analytes bind with immobilised antibodies (θ’’_SPR_). The resonance angle shift is θ’’_SPR_—θ’_SPR_.

Surface plasmon resonance (SPR) is a well-known sensing technology used for characterising kinetics of ligand–receptor interactions and these types of sensors offer unique real-time and label-free measurement capabilities with high detection sensitivity [44,45,46]. Figure 2A shows a typical SPR biosensor configuration where biorecognition elements, such as antibodies, are immobilised on the surface of a thin gold film. Surface plasmons are charge density oscillations that propagate along the metal-dielectric (sensing-medium) interface. They are excited by light (monochromatic) that is incident on the metal through a prism (in the prism coupling method) at a particular incident angle, called the resonance angle. Because some energy of the incident light is absorbed by the surface plasmons, the intensity of the reflected light is reduced at the resonance angle. When a sample in liquid form is allowed to flow across the sensor surface, the target analytes in the sample are captured by the immobilised biorecognition elements resulting in a change in the refractive index (RI) of the sensing medium, which shifts the resonance angle, as depicted in Figure 2B. This shift is proportional to the change in analyte mass on the gold film. In SPR imaging (SPRi), the reflected light is imaged using a charge-coupled device (CCD) [47,48]. In another type of SPR configuration known as localised SPR (LSPR), where the thin gold film is replaced by metallic nanoparticles, light incident on the nanoparticles excites coherent oscillations of the electron cloud around each particle, which is called a localised surface plasmon (LSP). Resonance occurs when the frequency of the light matches the LSP oscillation frequency. The analyte detection method is similar to conventional SPR [49,50].

#### 2.2.1. SPR/LSPR Biosensor for COVID-19

**Figure 3:**
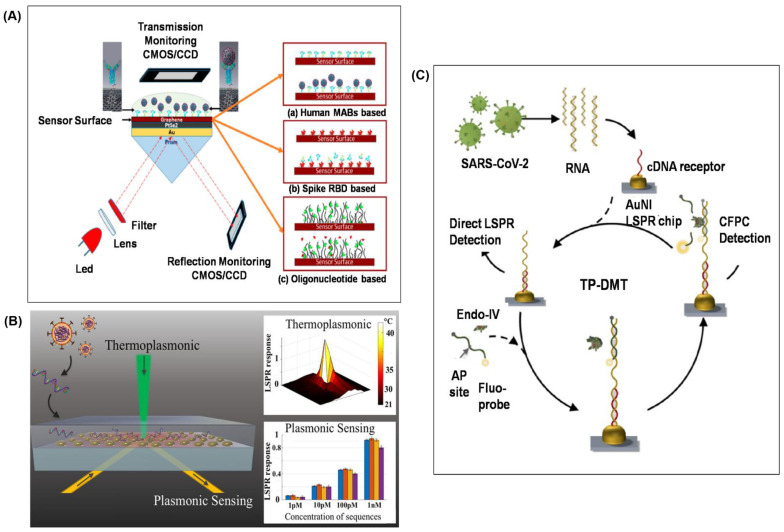
SPR and LSPR biosensor for SARS-CoV-2 detection. (**A**) Graphene-based multiple-layer coated (Bk_7_/Au/PtSe_2_/Graphene) SPR biosensor. Reprinted with permission from [35]. (**B**) Dual-functional PPT enhanced LSPR biosensing system. Reprinted with permission from [37]. Copyright 2020, American Chemical Society. (**C**) Thermoplasmonic-assisted dual-mode transducing (TP-DMT) biosensing system. Reprinted with permission from [38]. Copyright 2021, American Chemical Society.

SPR and LSPR technologies have been explored for detecting the SARS-CoV-2 virus. Recent reports on these types of sensors show promising sensitivity with fast and reliable detection. In one of the recent works, a two-dimensional heterostructure, PtSe_2_/Graphene, was attached to the gold film of the SPR sensor—the sensor configuration is shown in Figure 3A. The sensing region comprises three different ligand-analyte modes: (i) the monoclonal antibodies (mAbs) as ligand and the SARS-CoV-2 virus spike RBD as analyte, (ii) the virus spike RBD as ligand and the virus anti-spike protein (IgM, IgG) as the analyte and (iii) the specific RNA probe as ligand and the virus single-stranded RNA as analyte [35]. The sensor employed BK_7_ type prism glass, which generates stronger surface plasmon waves at the metal-dielectric interface. The hetero-structure (PtSe_2_/Graphene) provided an increased surface area for better adsorption of the target analyte, and hence enhanced sensitivity of the sensor. Graphene’s high conductivity, large surface area and chemical stability improved interaction between the target analyte and the ligand [51]. It was observed that the sensitivity tend to increase by (1 + 0.55) × L times for an increase of L number of graphene layers [35]. Using multiple graphene layers is promising in detecting different analytes such as virus spike RBD, antibodies (IgG or IgM) and viral RNA with a detection sensitivity of 183.33° RIU^−1^, 153.85° RIU^−1^ and 140.35° RIU^−1^ in SPR angle, respectively.

SPRi-based biosensors for detecting SARS-CoV-2 have also been developed. These sensors follow the principles of SPR and have been utilised to study binding kinetics between biomolecular species in the past [52,53,54]. SPRi assay was used for quantitative measurement of IgG, IgM and IgA antibodies binding to the RBD spike protein in serum sample [43]. RBD is an ideal target for blocking and neutralisation therapies. This is because viral infection occurs when the S protein on SARS-CoV-2 recognises and binds to the ACE2 receptor on the human host cell through its RBD [55]. Hence, this assay is ideally suited for monitoring the concentration of anti-RBD antibodies of both COVID-19 patients and healthy people who are vaccinated against SARS-CoV-2. 

Qiu and co-workers [37,38] demonstrated photothermal-assisted plasmonic sensing (PTAPS) for SARS-CoV-2 detection. Their biosensing device, as shown in Figure 3B, utilises LSPR for the detection of unamplified SARS-CoV-2 [37]. Two-dimensional gold nano-islands (AuNI) played the roles of nanoabsorber, nanoheater and nanotransducer. In this dual-functional system, AuNI functionalised with cDNA receptors were used to detect SARS-CoV-2 RNA through nucleic acid hybridisation. The entire AuNI sensing surface functionalised with a sufficient amount of thiol-cDNA receptor increased sensitivity and inhibit non-specific binding. Non-radiative decay of the resonantly-excited LSPs produces heat that is localised near the AuNI [56]. This plasmonic photothermal (PPT) effect aids the in-situ hybridisation of the RdRp-COVID sequence and its cDNA for SARS-CoV-2 detection. With the use of localised PPT heating, false positive is minimised as the imperfectly matched sequences have difficulty remaining attached to the probe. This LSPR sensor has a detection limit of 0.22 ± 0.08 pM but is highly specific in discriminating the SARS-CoV-2 sequence from similar RdRp-SARS sequence. 

Qiu et al. [38] extended the previous PTAPS method by introducing a novel concept of thermoplasmonic-assisted dual-mode transducing (TP-DMT), as shown in Figure 3C. This dual-mode system combines (1) an amplification-free direct viral RNA detection and (2) an amplification-based cyclic fluorescence probe cleavage (CFPC) detection to provide self-validating biosensing readout for quantifying the SARS-CoV-2 sequences within 30 min. The first LSPR signal was based on the hybridisation between the target viral sequences and the functionalised thiol-DNA receptors. The amount of captured sequence is directly proportional to the virus concentration in the sample, and the limit of detection (LOD) was as low as 0.1 ± 0.04 pM. In the CFPC detection, endonuclease IV (site-specific nuclease) was utilised to cleave the apurinic/apyrimidinic (AP)-site modified fluorescent probe from the target viral sequence under local PPT heating. The released fluorescent probe was used to quantify the concentration of the virus, which improved the LOD to 0.275 ± 0.051 femtomolar (fM). This dual-mode sensing method employed two interdependent yet different tests on the AuNI chip, and provides two sensitive readouts of viral sequence to yield remarkably low limits of detection in pM and fM, respectively. When the final readout of the direct viral hybridisation detection exhibited a weak response, the CFPC method was used for verification and it could even detect a trace amount of virus in the sample.

#### 2.2.2. Advantages and Limitations

The SPR-based biosensors we have reviewed for SARS-CoV-2 detection have good sensitivity and reusability. However, they lack selectivity and the refractive index of the biosensors are also affected by temperature, non-specific or specific adsorption on the sensor surface, and changes in buffer concentrations [57]. Hence, the sensor surface needs to be modified with a ligand (e.g., antibody, DNA probe) to selectively capture the target analyte.

On the other hand, SPR-based biosensors allow the integration of nanostructures or nanoparticles to enhance the detection sensitivity [58,59]. The extent of improvement depends strongly on the shape of the nanostructure and the type of metal used [49,59,60]. As mentioned in the previous section, two-dimensional heterostructure [35] and AuNIs [37,38] attached to the gold film in the sensor exhibited maximum sensitivity of 200° RIU^−1^ and detection of sample analytes at an ultra-low concentration in the range of pM and fM, respectively. SPR-based sensors allow the tailoring of the sensing medium to improve sensor performance and sensitivity by using multi-layers for the medium or altering the medium thickness [61,62].

However, plasmonic virus detection from clinical specimens is still limited due to surface fouling of the receptor surface and interference of non-specific bindings. Surface fouling limits the application of plasmonic biosensors by blocking recognition element immobilisation and specific binding [63]. It can be prevented by selecting the appropriate sample dilution ratio or the design of ultra-low fouling surfaces with anti-fouling strategies using polymer-based surface chemistry or zwitterionic technology [64,65].

Biosensors with good reusability are highly desirable because it lowers the cost per test. The solvent environment is a key parameter that determines the binding of antibody-antigen. The reversible non-covalent interaction between antibody and antigen can be disrupted by high salt concentration, extreme pH and detergents [66]. Hence, chemical regeneration is the most widely used approach to regenerate the sensor by chemically altering the solvent environment with a regeneration solution (e.g., acid/base, detergent, glycine or urea). SPR sensor could be chemically regenerated for SARS-CoV-2 detection [67]. 

### 2.3. Electrochemical Biosensor

**Figure 4:**
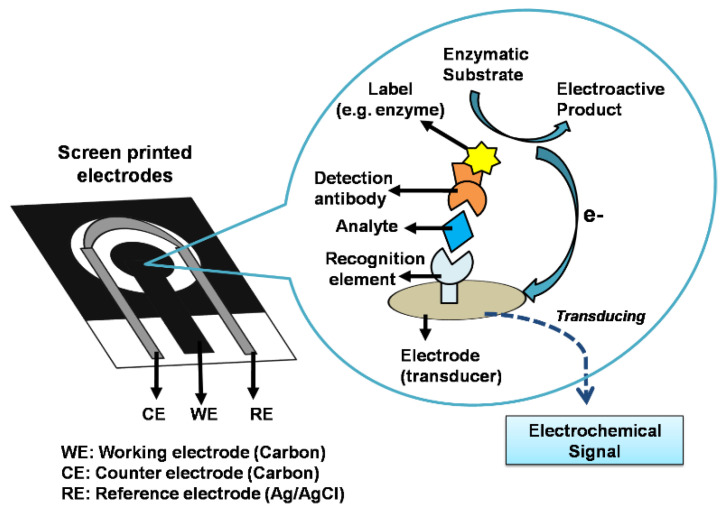
Screen-printed-electrode based electrochemical biosensor. Typical interactions on the working electrode among the biorecognition element, target analyte and detection antibody labelled with an enzyme. As shown, the enzyme oxidises an enzymatic substrate to produce an electroactive product that releases electrons, which are transduced as a measurable electrochemical signal.

Electrochemical biosensors are known for their small size, cost-effectiveness, ease of use and fast response. These types of sensors are extensively used in the development of point of care devices for diagnosing viral infections [68,69]. Among the electrochemical techniques, screen-printed electrode (SPE) technology has aided the development of portable sensors considerably as it provides miniaturised but robust and user-friendly electrodes at low production costs [70,71]. Figure 4 shows a typical configuration of an SPE-based electrochemical sensor employing a three-electrode system: a reference electrode, a counter electrode and a working electrode transduction element for biochemical reaction. The working electrode surface of SPE can be easily modified to immobilise specific biorecognition elements, such as an antibody to target the analyte. The antibody-antigen interaction on the electrode surface is transduced into a measurable electrical quantity. Alternatively, the catalytic reaction of a signal probe (e.g., enzyme), which labels the detection antibody, forms an electroactive product that releases electrons, which are transduced as a measurable electrochemical signal. The electrochemical measurement can be one of the following: (1) potentiometric-based, (2) amperometric-based, (3) conductometric or impedimetric-based [72,73] to determine the concentration of analyte in a sample. The SPE-based sensor can be integrated with a portable instrument (e.g., potentiostat) or reader (e.g., computer or a smartphone) [74] for digital processing.

Compared to conventional electrode materials (e.g., glassy carbon or carbon paste electrodes), SPEs have the advantage of cost, disposability, size and do not require electrode polishing prior to electrochemical detection. Because of its compact size, SPE only requires a small reagent volume for assay, as low as a few µL. This makes the SPE-based electrochemical biosensor a potential tool for on-site measurement in remote regions with limited resources. Moreover, the electroanalytical performance and sensitivity of the sensor can be enhanced by modifying the SPE working electrode surface with nanomaterials such as gold nanoparticles, graphene and carbon nanotubes to increase the electroactive area of the electrode for further immobilisation of biomolecules such as antibodies, protein or nucleic acid [75,76,77]. Graphene are two-dimensional carbon nanomaterials, in which the carbon atoms are positioned in a hexagonal honeycomb lattice [78], whereas carbon nanotubes are an allotropic form of carbon that can be rolled up into cylindrical tubes (e.g., single-walled or multi-walled) [79]. They possess characteristic properties of large surface area and excellent electrical conductivity. Hence, they are used as an electro modifier to promote electron transfer between a target analyte and electrode and thereby achieve high detection sensitivity [80,81].

#### 2.3.1. Electrochemical Biosensor for COVID-19

Rural communities bear a higher burden from the COVID-19 outbreak due to limited healthcare resources. The RT-PCR diagnostic method is costly and complicated in procedures, which makes it unsuitable for the low-resource area. Disposable electrochemical biosensors based on SPEs are promising alternatives for rapid, affordable, direct detection of SARS-CoV-2 at the point of care.

**Figure 5:**
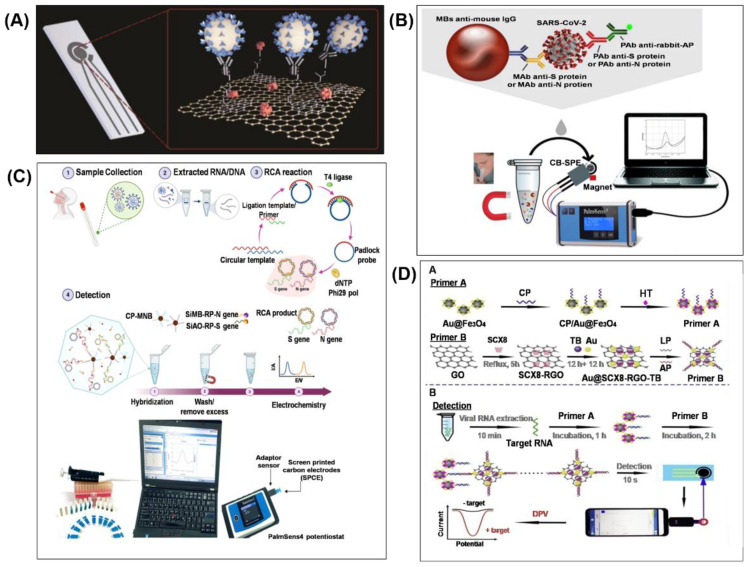
Electrochemical biosensor for SARS-CoV-2 detection. (**A**) Graphene-based electrochemical biosensor. Reprinted with permission from [39]. (**B**) MBs-based electrochemical biosensor. Reprinted with permission from [40]. Copyright 2020, Elsevier B.V. (**C**) Electrochemical biosensor with RCA of the N and S genes. Reprinted with permission from Lertanantawong, B (2021). Copyright 2021 Springer. (**D**) Smartphone-based supersandwich-type electrochemical biosensor. Reprinted with permission from [88]. Copyright 2021, Elsevier B.V.

Figure 5A shows a recent graphene-based SPE sensor, which is functionalised with a monoclonal anti-spike antibody for detecting SARS-CoV-2 spike antigen. The sensor detected spike protein in a saliva sample, by using square wave voltammetry (SWV) to measure the change of the ferri/ferrocyanide signal, in about 45 min. The ferri/ferrocyanide couple was used as a redox probe and the electrochemical detection was achieved by measuring the peak current of the ferro/ferricyanide redox after the biomolecular binding event on the sensing surface. The peak current is reduced because the target analyte prevented the redox probe from contacting the conductive surface [82]. The LOD of this sensor for spike protein was 20 µg mL^−1^. However, its sensitivity is lower compared to laboratory ELISA tests, which can detect as low as 3 ng mL^−1^ [39].

In another approach, magnetic beads (MBs)-based immunoassay is coupled with electrochemical detection for viral RNA and antigen detection with high sensitivity. MBs have the potential to reduce the incubation time from hours to minutes and is easily separated from a complex matrix under the action of an external magnetic field; therefore non-specific adsorption is almost negligible [83,84]. Fabiani et al. presented an electrochemical biosensor by combining MBs-based immunoassay and SPE, modified with carbon black, with a portable potentiostat as a reader for SARS-CoV-2 detection in saliva (Figure 5B) [40]. In the MBs-based immunoassay, target protein (S or N protein) in the untreated saliva sample was selectivity captured by the respective monoclonal antibody–bound MB and sandwiched by the respective polyclonal antibody. A secondary antibody attached to the polyclonal antibody is labelled with an alkaline phosphate enzyme. In the electrochemical detection, the labelled beads were drop cast on the working electrode of SPE with 1-naphthyl phosphate to induce the formation of enzymatic by-product 1-napthol. The enzymatic reaction (enzymatic by-product) was electrochemically measured via differential pulse voltammetry (DPV) using a portable potentiostat. The sensor was tested using 24 clinical samples (positive and negative); the true positive rate was 100% and the true negative rate was 88.2% for detecting the S protein. The sensor required 30 min for reaction and exhibited the lowest detection limit of 19 ng mL^−1^ for S proteins and 8 ng mL^−1^ for N proteins, respectively. However, repeated incubation and washing steps are required to perform the MBs-based immunoassay prior to the electrochemical detection.

Chaibun et al. developed an ultrasensitive electrochemical biosensor (as shown in Figure 5C) based on multiplex isothermal rolling circle amplification (RCA) for rapid detection of viral N and S genes of SARS-CoV-2 [36]. The sensor works on a one-step strategy comprised of mixing capture probe-conjugated magnetic bead particle (CP-MBs), silica reporter probe (silica nanoparticles coated redox dye), and the target (viral N and S genes) in a single hybridisation step, followed by a single washing step. The sandwich hybridisation of RCA amplicons with probes that are functionalised with redox-active labels (e.g., methylene blue for N gene and acridine orange for S gene) was detected by DPV. The redox-active reaction could detect as low as 1 copy μL^−1^ of viral N or S genes in less than 2 hours. Moreover, the use of magnetic capture and separation of targets from non-targets reduces the chance of residual contamination and pipetting error, thereby reducing the risk of erroneous results and improving the assay precision [85]. The potentiostat is a key component for reading electrochemical signals, but the traditionally large and expensive bench-top versions limit its use in resource-limited environments. Hence, a portable potentiostat was developed and used, in conjunction with a computer, for electrochemical measurement at the point of use. 

The smartphone is the most widely used portable device in the world. It is equipped with powerful connectivity features and researchers have explored the use of smartphones as a wireless diagnostic tool [74,86]. A portable potentiostat with wireless connectivity to a smartphone would facilitate electrochemical analysis at the point-of-use, where access to a computer or wired connection to a device is difficult or impossible [87]. A smartphone-based supersandwich-type electrochemical biosensor was demonstrated as shown in Figure 5D for SARS-CoV-2 RNA detection without nucleic acid amplification and reverse-transcription [88]. The assay employed for sensing utilised two different kinds of primer A and B. The former contains Au@Fe_3_O_4_ nanocomposites, the capture probe (CP) and 1 mM hexane-1-thiol (HT). The latter contains Au@p-sulfocalix[8]arene(SCX8)-toluidineblue(TB)-graphene(RGO) nanocomposites, the labelled probe (LP) and the auxiliary probe (AP). Primer A and then B are mixed with the target for 1 h and 2 h, respectively. Subsequently, the sandwich structure formed was dropped on the SPE for electrochemical measurement of TB signal by a smartphone in less than 10 s. TB is a basic thiazine metachromatic dye with a high affinity for nucleic acids, thereby staining tissues with a high DNA and RNA content [89]. This method requires a long incubation time of about 3 h for the formation of sandwich structure and more than 12 h for the preparation of primer A and B. For detection of SARS-CoV-2 in clinical specimens, the true positive rate was 85.5% and 46.2% for confirmed and recovered patients, respectively. The sensor does not require RNA amplification and it only requires two copies of SARS-CoV-2 for an assay, which is advantageous compared to the existing PCR-based RNA assay. The detection limit of the tested samples was found to be 200 copies mL^−1^.

**Figure 6:**
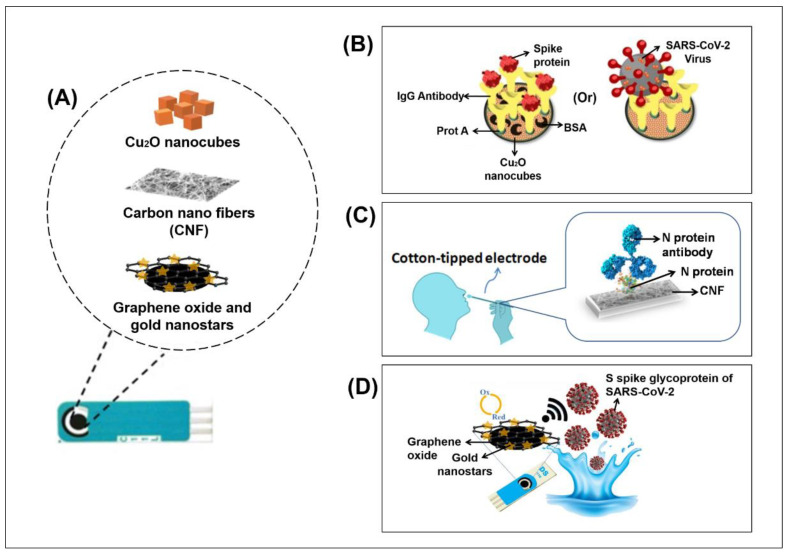
(**A**) Surface modification of screen printed electrodes with nanostructured materials (**B**) Electrochemical immunosensor with Cu_2_O nanocube coating for the detection of SARS-CoV-2 spike proteins. Bovine Serum Albumin (BSA) is used as a blocking agent. Reprinted with permission from Roushani, M (2021). Copyright 2021 Springer. (**C**) Cotton-tipped electrochemical immunosensor for the detection of virus nucleocapsid (N) protein. Reprinted with permission [91]. Copyright 2021, American Chemical Society. (**D**) Electrochemical diagnostic kit for the detection of SARS-CoV-2 S spike glycoproteins. Glycoproteins can be traced through the oxidation signals of gold nanostars, which are deposited on the surface of the modified electrode. Reprinted with permission [92]. Copyright 2021, Elsevier B.V.

A few other recently reported works utilised modified screen-printed electrodes with nanostructured materials, as depicted in Figure 6A, to enhance the detection of SARS-CoV-2 spike protein and glycoprotein. In [90], an electrochemical biosensor was fabricated using disposable carbon-based SPE, where the electrode surface was modified with Cu_2_O nanocubes. IgG anti-SARS-CoV-2 was attached to staphylococcal protein A (Prot A) that was loaded on the surface (Figure 6B). The larger surface area of the nanocubes provided more active sites to bind IgG and hence allowed more SARS-CoV-2 to be detected. The biosensor exhibited a LOD of 0.04 fg mL^−1^, and it was 100% successful in detecting true positive and negative samples, based on 16 clinical samples. In [91], the carbon electrode surface was modified with carbon nanofibers (CNF), followed by immobilisation of N protein and antibody for N protein, as depicted in Figure 6C. The CNF not only increased the surface area for more active sites for binding but also allowed a direct collection of nasal samples. The LOD of the sensor was 0.8 pg mL^−1^ for SARS-CoV-2 N protein antigen. In [92], the SPE surface was modified with graphene oxide and gold nanostars, and S spike glycoproteins were used to detect SARS-CoV-2 (Figure 6D). Based on 100 clinical samples, the LOD was 1.68 × 10^−22^ μg mL^−1^ and the true positive and negative rate was 95% and 60%, respectively.

#### 2.3.2. Advantages and Limitations

The reviewed electrochemical sensors based on SPEs for SARS-CoV-2 detection show excellent portability, with true positive rates ranging between 46.2% and 100%. Typically, commercially available SPEs were used as they are easy to use and disposable. The most commonly used material for the assembly of the working electrode of SPE is carbon, gold and platinum ink. SPEs modified with a range of nanoparticles or nanomaterials are also commercially available. The modification of the sensing surface with a large active surface area (e.g., gold nanoparticles, graphene, carbon nanotubes) increases the number of immobilised biorecognition elements and thus the number of available analyte binding sites, which increases the detection sensitivity of the biosensor. Compared to conventional glassy carbon or carbon paste electrodes, SPEs do not require electrode polishing and electrochemical pre-treatment by electro-deposition. Furthermore, potentiostats are already available in miniaturised formats, which enable on-the-spot or point-of-care applications.

At present, saliva tests offer a promising alternative to nasopharyngeal swabs for COVID-19 diagnosis, since collecting saliva is non-invasive and easy to self-administer [93,94]. However, differences in the sample collection methods—such as cough out (without sputum), split (exclude bubbles) or drooling—can affect the salivary composition and sensitivity to SARS-CoV-2. Consequently, the matrix effect may result in an inaccurate measurement for viscous saliva without any pre-treatment (e.g., dilution with phosphate buffer). However, high dilution of saliva samples with low viral content may lead to a false-negative result. Recently, spike protein detection in saliva samples using an electrochemical biosensor was proposed in [39,40]. It was suggested in [40] that using fresh saliva sampled after drinking a glass of water obviates the need for sample pre-treatment.

### 2.4. Field Effect Transistor (FET) Biosensor

**Figure 7:**
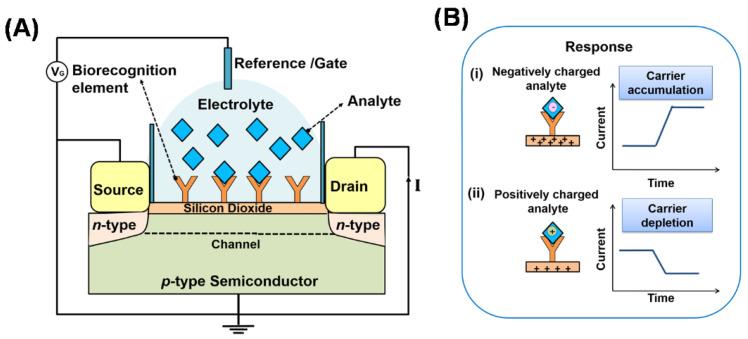
(**A**) Field-effect transistor (FET)-based biosensor with a *p*-type semiconductor channel. (**B**) (**i**) Negatively charged target molecules captured by the biorecognition elements accumulate positive charge carriers (holes) in the channel, which increases the current from the source to the drain electrode. (**ii**) If positively charged target molecules bind with the biorecognition elements, the holes are depleted and the current decreases.

As shown in Figure 7A, a typical FET-based biosensor consists of a semiconductor substrate with three terminals: (1) the source, (2) the drain and (3) reference or gate in contact with an electrolyte. The source and drain terminals are attached to the semiconducting substrate and a thin oxide layer (insulator) is deposited between these two terminals. Generally, biorecognition elements such as antibodies are immobilised on the oxide layer (sensor surface) to complete the biosensor construction. When a bias voltage is applied across the gate, an electric field is generated and charge carriers flow in the semiconductor channel from the source electrode to the drain electrode. The direction of current depends on the type of channel, either *p*-type or *n*-type [95]. The target molecules (e.g., protein, nucleic acid) usually carry charges and they will affect the current when they are bound to the recognition elements immobilised on the sensor surface. The current can thus be monitored as a function of time to detect the target molecules. For the *p*-type channel, negatively charged target molecules captured by the biorecognition elements accumulate positive charge carriers (holes) in the channel, which increases the current as depicted in Figure 7(Bi). Conversely, if positively charged target molecules bind with the biorecognition elements, the holes are depleted and the current is decreased as depicted in Figure 7(Bii). Different types of nanomaterials can be deposited on the sensor surface, such as silicon nanowires [96,97], carbon nanotubes [98,99,100] and graphene [42,101,102,103], for detecting a variety of biological analytes such as proteins, nucleic acids, ions and small molecules, respectively.

#### 2.4.1. FET Biosensor for COVID-19

Attachment of carbon-based two-dimensional materials and nanotubes (CNTs) on the surface of FET biosensors have been explored to enhance the detection of the target analyte. CNTs and graphene-based FETs have shown high sensitivity in detecting small concentrations of target analyte [104,105].

**Figure 8:**
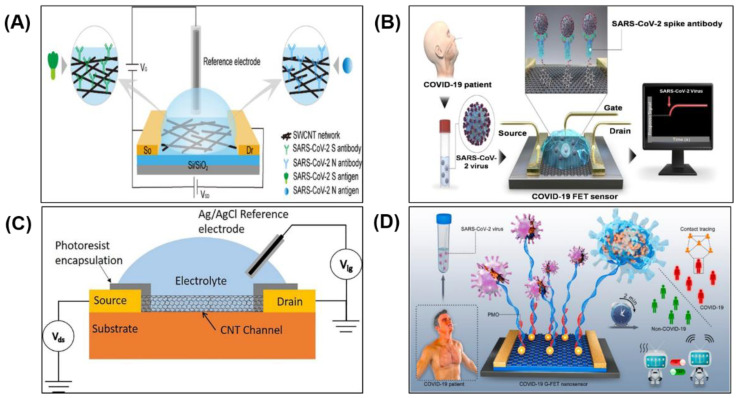
Field-effect transistor biosensor for COVID-19 detection. (**A**) SWCNT-based FET biosensor. Reprinted with permission from [42]. Copyright 2020, American Chemical Society. (**B**) Graphene-based FET biosensor. Reprinted with permission from [99]. Copyright 2021, American Chemical Society. (**C**) CNT-based FET fabricated on a flexible Kapton substrate. Reprinted with permission from [100]. Copyright 2021, Elsevier Ltd. (**D**) PMO-functionalised Graphene-FET biosensor. Reprinted with permission from [106]. Copyright 2021, Elsevier B.V.

Recently, a FET biosensor was developed by depositing SARS-CoV-2 spike protein and anti-nucleocapsid protein antibodies functionalised single-walled carbon nanotube (SWCNT) on the sensor surface to detect complimentary SARS-CoV-2 antigens (Figure 8A) [99]. Based on 28 positive samples, the SWCNT-based FET biosensor exhibited a true positive rate of 82.1% and 53.6% for detecting spike protein and nucleocapsid protein, respectively. Based on 10 negative samples, the true negative rate was 70% for both proteins. Overall, the SWCNT-based FET sensor exhibited excellent sensitivity with a LOD of 0.55 fg mL^−1^ and 0.016 fg mL^−1^ in detecting spike protein and nucleocapsid protein, respectively [99]. The sensor has the potential to be used as a rapid SARS-CoV-2 antigen test using clinical nasopharyngeal samples, without pre-processing, in less than 5 min.

In another approach, SARS-CoV-2 spike protein antibodies were functionalised on graphene sheets, which were then deposited on the sensor surface. This graphene-based FET biosensor is depicted in Figure 8B. The sensor was able to detect the SARS-CoV-2 spike antigen protein down to fg mL^−1^ in phosphate-buffered saline samples or clinical transport medium and with a LOD of 2.42 × 10^2^ copies mL^−1^ in clinical samples, at a response rate between 0 and 400 s [42].

However, for viral detection of SARS-CoV-2, the RNA probe is required to detect the presence of complementary nucleic acid sequences (target sequences). To fulfil this need, recently a liquid gated CNT network FET was fabricated on a flexible Kapton film [100], as shown in Figure 8C. The sensor was able to selectively detect a portion of the SARS-CoV-2 RNA through hybridisation. Here, the reverse sequence of the RNA-dependent RNA polymerase gene of SARS-CoV-2 was immobilised onto the CNT sidewalls. The RNA hybridisation was used as the primary signal generator and the liquid gated CNT network FET was used as the signal transducer. The biosensor showed a selective sensing response to the target sequence with a LOD of 10 fM.

In another remarkable FET biosensor, gold nanoparticles (AuNP) decorated graphene sheets and phosphorodiamidate morpholino oligos (PMO) probes were employed on the sensor surface for ultrasensitive and rapid SARS-CoV-2 detection (Figure 8D) [106]. The PMO probe is usually 25 bases in length, and they bind to complementary sequences of RNA or single-stranded DNA according to standard nucleic acid base-pairing [107]. The PMO enabled direct detection of SARS-CoV-2 RNA through the hybridisation between the PMO probe and SARS-CoV-2 RdRp without the need for further PCR amplification. For clinical readiness, an FET biosensor needs to have good reusability [108]. For SARS-CoV-2 RdRp detection, this sensor can be chemically regenerated for reuse by denaturing the PMO-RNA duplex with 8.3 M urea solution for 5 min. The high surface-area-to-volume ratio of AuNP, high conductivity of graphene, and high density of neutral PMO immobilisation provided more responsive testing than using charged oligonucleotide capture probe (e.g., single-stranded DNA probe) [106] for RNA detection. The sensor accuracy was analysed with a receiver operating characteristic (ROC) curve and it achieved an AUC of 0.995.

#### 2.4.2. Advantages and Limitations

Overall, the FET-based sensor provides fast detection and low LOD and does not require additional procedures for labelling during sample preparation. It is low cost, small in size and simple to operate. Nevertheless, the device sensitivity could be further improved, especially at a high concentration of sample analyte as the total number of antibodies anchored on the surface is limited by the small sensor size [109]. Non-specific interaction and screening effect could also diminish response and sensitivity [110]. To prevent non-specific binding, a blocking reagent (e.g., BSA or polyethylene glycol) is required to block the un-reacted active sites, or a covalent coupling (e.g., amide bond) is required to attach the antibody to the sensor [111,112].

**Figure 9:**
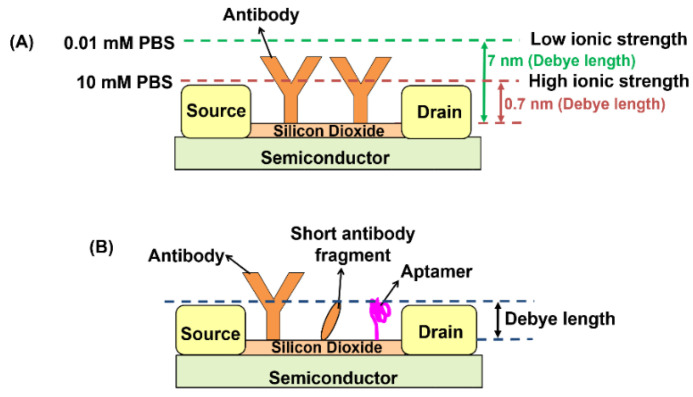
(**A**) The Debye length increases with the reduction of buffer ionic strength. (**B**) Short antibody fragments or aptamers could be used to bring the analyte-binding closer to the sensor surface and within the Debye length.

The Debye length problem remains an entrenched obstacle [113,114]. A layer of ions in the electrolyte above the sensor surface effectively ‘shields’ or ‘screens’ the analytes from the charge carriers in the semiconductor channel. The thickness of this layer is called the Debye length [111]. As shown in Figure 9A, the Debye length under physiological conditions (in 10 mM phosphate buffer saline (PBS) solution) is close to 0.7 nm [115], which is smaller than the size of antibody receptor molecules immobilised on the sensor surface, which are generally 10–15 nm in size [114]. It is difficult to detect analyte-binding that are beyond the Debye length in the physiological environment. There have been attempts to address this problem by using short antibody fragments (e.g., nanobody receptor) or aptamer (Figure 9B), which enable the analyte to bind closer to the sensor surface and achieve a detection limit down to the sub-picomolar range without the loss of selectivity [116].

The Debye length is proportional to the reciprocal of the ionic strength. To increase the Debye length, the electrolyte ionic strength can be lowered by diluting the sample, but this, in turn, dilutes the analyte concentration and may cause a change in protein structure, resulting in the loss of protein activity and binding affinity as well. For example, in [41], the 10 mM PBS buffer was diluted to 0.01 mM, which increased the Debye length from 0.7 nm to 7 nm, comparable to the size of the positive-charged spike protein S1 subunit antibody (7–10 nm). However, dilution makes it difficult to detect low-abundance analytes [117,118].

**Table 1:** Comparison of the latest developed biosensor: (a) SPR and LSPR, (b) Electrochemical and (c) FET for SARS-CoV-2 detection. In each row, ^(i), (ii), (iii)^ represent types of target analyte; ^1, 2, 3^ represent types of testing sample; ^a^ represents True Positive Rate (%) and ^b^ represents True Negative Rate (%).

**(a)**	**Type of Biosensor**	**Sensing Area & Recognition Element**	**Target Analyte**	**Testing Sample**	**Assay Time**	**Sensitivity**		**^a^ True Positive Rate (%)** **^b^ True Negative Rate (%)**	**Sample Confirmation Method**	**Number of Clinical Samples**	**Ref.**
						**Limit of Detection (LOD)**	**Detection Sensitivity** **(in SPR Angle)**				
	SPR	Graphene-based multiple-layer (BK_7_/Au/PtSe_2_/Graphene) modified with specific ligands	^(i)^ Spike RBD ^(ii)^ Anti-spike protein (IgG or IgM) ^(iii)^ Virus single-stranded RNA	Nasopharyngeal swabs and blood	−	−	^(i)^ 183.3° RIU^−1^ ^(ii)^ 153.85° RIU^−1^ ^(iii)^ 140.35° RIU^−1^	−	Not available	Not available	[35]
		Surface Plasmon Resonance imaging (SPRi)	IgG, IgM and IgA	Serum	−	−	−	−	RT-qPCR	384 sera	[43]
	LSPR	Gold nanoislands (AuNIs) functionalised with complementary DNA receptor	SARS-CoV-2 sequences	Multigene mixture (RdRp, ORF1ab, and E gene)	−	0.22 ± 0.08 pM	−	−	Not available	Not available	[37]
			SARS-CoV-2 sequences	Nasopharyngeal swabs	30 min	0.1 ± 0.04 pM (Direct viral sequence detection) 0.275 ± 0.051 fM (CFPC detection)	−	−	RT-PCR	8 samples (5 positive and 3 negative samples)	[38]
**(b)**	**Type of Biosensor**	**Sensing Area & Recognition Element**	**Target Analyte**	**Testing Sample**	**Assay Time**	**Sensitivity**		**^a^ True Positive Rate (%)** **^b^ True Negative Rate (%)**	**Sample Confirmation Method**	**Number of Clinical Samples**	**Ref.**
						**Limit of Detection (LOD)**					
	Electrochemical	Graphene-based SPE functionalised with a monoclonal anti-spike antibody	Spike protein	Saliva	45 min	20 µg mL^−1^		−	Not available	Not available	[39]
		Antibodies for S or N proteins immobilised on magnetic beads (MBs)	^(i)^ Spike protein ^(ii)^ Nucleocapsid protein	Untreated saliva	30 min	^(i)^ 19 ng mL^−1^ ^(ii)^ 8 ng mL^−1^		^a (i)^ 100% ^b (i)^ 88.2%	RT-PCR	24 samples (7 positive and 17 negative samples)	[40]
		Sandwich hybridisation of RCA amplicons with probes functionalised with redox-active labels	^(i)^ S gene ^(ii)^ N gene	Nasopharyngeal swabs	<2 h	1 copy μL^−1^		^a (i)^ 100% ^b (i)^ 100% ^a (ii)^ 100% ^b (ii)^ 100%	qRT-PCR	106 sample (41 positive and 65 negative samples)	[36]
		p-sulfocalix[8]arene functionalised graphene (SCX8-RGO)	SARS-CoV-2 RNA	Throat swabs	<10 s	200 copies mL^−1^		^a^ 85.5% (confirmed patient); 46.2% (recovered patient)	RT-qPCR	88 RNA extracted from 25 SARS-CoV-2-confirmed patients and 8 recovered patients	[88]
		Cu_2_O nanocubes based SPE immobilised with IgG anti-SARS-CoV-2 spike antibody	Spike protein	^1^ Nasopharyngeal swabs ^2^ Saliva	<20 min	0.04 fg mL^−1^		^a (1)^ 100% ^b (1)^ 100% ^a (2)^ 100% ^b (2)^ 100%	PCR	16 samples (8 positive and 8 negative samples)	[90]
		Carbon nanofiber-based SPE functionalised with nucleocapsid antigen	Nucleocapsid protein	Nasopharyngeal swabs	20 min	0.8 pg mL^−1^		−	RT-PCR	3 samples (2 positive and 1 negative samples)	[91]
		Graphene oxide-based SPE with 8-hydroxyquinoline and gold nanostars	Viral spike glycoproteins	Nasopharyngeal swabs	1 min	1.68 × 10^−22^ μg mL^−1^		^a^ 95% ^b^ 60%	RT-PCR	100 samples (60 positive and 40 negative samples)	[92]
**(c)**	**Type of Biosensor**	**Sensing Area & Recognition Element**	**Target Analyte**	**Testing Sample**	**Assay Time**	**Sensitivity**		**^a^ True Positive Rate (%)** **^b^ True Negative Rate (%)**	**Sample Confirmation Method**	**Number of Clinical Samples**	**Ref.**
						**Limit of Detection (LOD)**					
	FET	Single-walled carbon nanotube (SWCNT) functionalised with anti-SARS-CoV-2 spike protein antibody and anti-nucleocapsid protein antibody	^(i)^ Spike protein and ^(ii)^ Nucleocapsid protein	Nasopharyngeal swabs	<5 min	^(i)^ 0.55 fg mL^−1^ ^(ii)^ 0.016 fg mL^−1^		^a (i)^ 82.14% ^a (ii)^ 53.57% ^b (i)(ii) ^ 70%	PCR	38 samples (28 positive samples and 10 negative samples)	[99]
		Graphene channel functionalised with SARS-CoV-2 antibody	SARS-CoV-2 RNA	Nasopharyngeal swabs	>1 min	2.42 × 10^2^ copies mL^−1^		−	RT-PCR	3 SARS-CoV-2-confirmed patients	[42]
		Carbon nanotube channel immobilised with the reverse sequence of the RNA-dependent RNA polymerase gene of SARS-CoV-2	SARS-CoV-2 RNA	Buffer	−	10 fM		−	Not available	Not available	[100]
		Phosphorodiamidate morpholino oligos (PMO) probe immobilised on the AuNP surface	SARS-CoV-2 RNA	^1^ Buffer, ^2^ Throat swab ^3^ Serum	2 min	^1^ 0.37 fM ^2^ 2.29 fM ^3^ 3.99 fM		−	RT-PCR	30 throat swab samples from 20 SARS-CoV-2-confirmed patients and 10 excluded individuals.	[106]

## 3. Strategies to Enhance the Biosensor Performance

The accuracy of the reviewed biosensors in detecting negative samples are short of the WHO minimum of 97%. This can be improved by choosing biorecognition elements that are highly selective in binding with the target analytes and hence cross-reactivity with unrelated molecules can be avoided to reduce false positives. Moreover, the true positive rate (ranging from 46.2% to 100%) of the biosensors do not all meet the WHO minimum of 80%. This can be improved by increasing the surface area of the sensor site using surface modification techniques to enhance the sensor sensitivity to a low concentration of bioanalytes. These enhancement strategies for COVID-19 biosensor performance are elaborated in the following sections.

### 3.1. Potential Biorecognition Elements

In general, the detection technique used in biosensors can be classified as labelled or label-free. Labelled biosensors commonly rely on specific labels, such as enzymes (e.g., horseradish peroxidase (HRP), alkaline phosphatase (ALP)), fluorescent molecules or electroactive compounds, to detect analytes. The label helps in signal amplification and increase sensing selectivity, but at the same time, it increases the overall sensor cost and response time. On the other hand, label-free biosensors rely on biorecognition elements to directly detect different types of analyte targets ranging from proteins to DNA and viruses. There is a wide range of biorecognition elements—such as enzyme, antibody, nucleic acid, aptamers, molecularly imprinted polymers—with unique characteristics for the interaction with a specific target of interest [119,120].

In COVID-19 biosensors, antibodies and nucleic acid probes are the most common biorecognition elements used for recognising SARS-CoV-2 biomarkers (e.g., viral proteins, human immunoglobulins or viral RNA) through the formation of antigen-antibody immunocomplex and single-stranded-DNA/oligonucleotide complementary strands complexes, respectively.

In general, monoclonal antibodies are preferred for assay involving binding of a specific antigen. These antibodies are specific to a single epitope of a target molecule, but a slight change in conformation may lead to a dramatically reduced binding capacity [121,122]. However, an alternative approach known as antibody phage display can be employed, where the monoclonal antibodies can be engineered to improve binding affinity with specific viral markers [123,124]. The conventional method of producing monoclonal antibodies requires expensive experimentation with animals and labour-intensive procedures. Besides high cost, the antibodies have a limited lifespan and are susceptible to high temperature [125,126].

Alternatively, high affinity and specificity aptamers have emerged as a substitute to monoclonal antibodies as biorecognition elements in biosensing. Aptamers are artificial single-stranded RNA or DNA oligonucleotides that can be chemically synthesised by an in vitro selection process called Systematic Evolution of Ligands by Exponential Enrichment (SELEX) [127]. Similar to antibodies, aptamers can bind specifically to the target based on structural recognition [127]. Furthermore, aptamers offer many advantages over antibodies, including smaller size, long shelf-life, stable to changes in pH, temperature, and ionic strength [128]. Compared to antibodies, aptamers have a much lower molecular weight (6–30 KDa, 2 nm) than antibodies (150–180 kDa, 15 nm), which allows them to bind with a wide range of potential targets such as ions, small molecules, viruses, and proteins [129,130,131,132]. Aptamers also have high thermal stability (up to 95 °C), and they are suitable for repeated use as they can be regenerated easily after denaturation [133]. In particular, when used as a biorecognition element in FET biosensors, the smaller size and compact structure of the aptamers allow the binding event to take place within the Debye length, thus overcoming the Debye length limitation. The bound targets are located closer to the sensor surface, and therefore stronger electrical signal is transduced from the binding event [134].

Phosphorodiamidatemorpholino oligomers (PMO) are uncharged analogues of nucleic acids. Compared to natural nucleic acid probes, the PMO probe offers better stability and higher binding strength owing to its neutral character [135]. RNA and DNA probes are negatively charged in physiological conditions due to their phosphate groups on the nucleic acid backbone. Therefore, electrostatic repulsion exists between the highly negative charged probe and target sequence, which decreases the hybridisation efficiency. Hence, RNA and DNA probes require high ionic strength conditions to shield their intermolecular repulsive force for hybridisations [136]. However, PMO probes have no net charge and they are insensitive to the ionic strength of the buffer, so they can retain their excellent binding affinity to target nucleic acid under the condition of low or high ionic strength [137]. 

### 3.2. Potential Nanomaterials for Sensor Surface Modification

With the rapid development of nanotechnology in the past few years, various nanomaterials, such as gold nanoparticles, graphene and carbon nanotubes, have been widely used in the design of biosensors as transduction substrates to enhance sensing performance. Nanomaterials characteristics, such as nano-scale size, larger surface area and higher conductivity, aid in signal amplification [138]. Besides that, the inherent large surface areas of nanomaterials can enhance the loading effect caused by the bioreceptor [139], thereby improving the sensitivity and stability of the biosensor.

In SPR-based sensors, metal nanoparticles (e.g., gold nanoparticles) have been commonly incorporated on the sensor surface [140,141]. As nanoparticles possess unique physical, electronic and chemical properties, large surface area and high free surface energy, biomolecules (e.g., antibody) are strongly adsorbed onto the surface. This improved the SPR detection limit from the nM range to the pM range [142]. The sensitivity improvement by nanomaterials also depends on the type of structures such as nanoshells, nanospheres, nanorods and nanowires. Compared to other shapes, high aspect ratio nanorods (NRs) offer a higher sensitivity to refractive index changes [143,144]. When NRs are used in a sandwich assay format, concentrations in the fM range can be detected [145,146]. In another strategy, dual nanomaterials (e.g., NRs and quasi-spherical nanoparticles (qsNPs)) have yielded attomolar range (aM) detection, which is about a 10-fold enhancement in sensitivity compared to sensor employing a single type of nanoparticles [141].

In addition, two-dimensional materials such as graphene are widely used in SPR, FET and electrochemical based biosensors. The high surface area to volume ratio and carbon-based ring structures of graphene enhances the adsorption of biorecognition elements onto the sensor surface [147,148]. Adding graphene-based materials on the gold film of SPR sensors allow detection down to fg/mL of mass change [149]. Graphene and reduced graphene oxide possess excellent electronic properties, which allow them to be used as sensor surface material in FET [150] and electrochemical sensors [151]. Due to the excellent electronic and adsorption properties of these materials, the LOD of the FET biosensor reported in [147] is 1 fM and 10 fM for the detection of the Japanese encephalitis and avian influenza virus, respectively. However, graphene yields a small current on-off ratio in FETs, which limits its sensitivity [136]. A large on-off ratio is required to reduce static leakage current for the sensor. To achieve this, two-dimensional semiconducting transition metal dichalcogenides (TMDCs) (e.g., molybdenum disulfide (MoS2) nanoflakes, tungsten disulfide (WS_2_) nanosheets) and black phosphorus (BP) can be explored. TMDCs have a larger bandgap than graphene and excellent characteristics such as large surface area and high electron transfer [152,153]. BP has adjustable band gap value and high carrier mobility [154,155].

## 4. Conclusions

The recently developed SPR-based, electrochemical and FET-based biosensors for SARS-CoV-2 detection we have reviewed are promising alternatives to currently available point of care (POC) tests. However, in addition to the limitations we have highlighted, none of the sensors met the WHO minimum requirement for true positive and true negative detection rates. Some of the sensors met the former requirement, but none met the latter requirement. The detection accuracies (particularly the true negative/false positive rate) need to be significantly improved before the sensors could be translated into POC devices for commercial use. The biorecognition elements and sensor surface modification materials we have suggested could be explored to improve the true negative and true positive rate, respectively.

## Data Availability

Not applicable.
